# Phenolics profile and anti-proliferative activity of *Cyphomandra Betacea* fruit in breast and liver cancer cells

**DOI:** 10.1186/s40064-016-3777-x

**Published:** 2016-12-20

**Authors:** Maisarah Abdul Mutalib, Faisal Ali, Fauziah Othman, Rajesh Ramasamy, Asmah Rahmat

**Affiliations:** 1 Department of Nutrition and Dietetics, Faculty of Medicine and Health Sciences, Universiti Putra Malaysia (UPM), 43400 Serdang, Selangor Malaysia; 2 Haematology Department, University Hospital, Faculty of Medicine and Health Sciences, Sana’a University, Sana’a, Yemen; 3 Department of Human Anatomy, Faculty of Medicine and Health Sciences, Universiti Putra Malaysia (UPM), 43400 Serdang, Selangor Malaysia; 4 Department of Pathology, Faculty of Medicine and Health Sciences, Universiti Putra Malaysia, 43400 Serdang, Selangor Malaysia

**Keywords:** Anti-proliferative activity, *Cyphomandra betacea*, Breast cancer cell, Liver cancer cell, Phenolics

## Abstract

**Background:**

*Cyphomandra betacea* (C. *betacea*) belongs to the Solanaceae family. This study was aimed to evaluate the anti-proliferative of *C.betacea* crude extract against selected cancer cell lines (breast and liver cancer) and to identify the polyphenolics profile (phenolic acids and flavonoids) of *C*. *betacea* fruits. Anti-proliferative effect of the extracts was examined using MTT (3-(4,5-dimethylthiazol-2yl)-2,5-diphenyltetrazolium bromide) assay, followed by cell morphology analysis using acridine orange and propidium iodide double staining.

**Results:**

The phenolics profile was characterized using high-performance liquid chromatography (HPLC). *C*. *betacea* extract showed a high cytotoxic effect against liver and breast cancer cell lines with the IC_50_ value of 30 and 80 µg/ml, respectively. Phenolics profiling showed that *C*. *betacea* extract has a wide variety of polyphenolic compounds which are the responsible substances underlying the cytotoxic activity. The microscopic examination analysed by acridine orange and propidium iodide double staining showed that viable cells of the liver and breast cells were well rounded, large and intact green nuclei compared with the treated cells that characterized by apoptotic features (shrunken nuclei with a less quantity of cytoplasm).

**Conclusion:**

The present study demonstrated that the anti-proliferative properties of *C*. *betacea* fruits were partially attributed to the rich phenolics content. It supports the hypothesis that *C*. *betacea* fruits have potential as an effective agent in cancer therapy.

## Background

It is known that some of the medicinal plants possess anticancer activity. In addition, polyphenols from fruits or vegetables are the responsible molecules for the chemopreventive effects. Epidemiological studies have shown that high dietary intake of polyphenols from plant is associated with a decreased risk of the degenerative diseases, including cardiovascular diseases and certain cancers (Kumar and Pandey 2013). The major dietary polyphenols are flavonoids and the most anticancer compounds of which are catechins, curcumin, resveratrol and genistein (Dai and Mumper 2010). The process of carcinogenesis includes 3 important stages; initiation, promotion and progression (Wattenberg 1985). These phenolics substances have been shown to hinder cancer progression by interfering with each of the stage of carcinogenesis. Several studies have proposed that the anticancer ability of some dietary polyphenols such as quercetin, genistein, apigenin was depending on the inhibition of the proliferation process of various cancers in vitro and in vivo (Fantini et al. 2015).

Phenolic acids are non-flavonoid polyphenolic compounds that are often in bound form which are abundant in fruits, vegetables, grains and seeds (Tsao 2010). They are commonly represented by vanillic acid, *p*-coumaric acid, caffeic acid, ferulic acid and gallic acid. Caffeic acid has been reported to suppress the activation of NF-κB and AP1 which might contribute to their chemopreventive effects (Bharti and Aggarwal 2002). Flavonoids are further divided into several classes which include flavones, flavanones, isoflavones, anthocyanins, flavonols, and flavanols. Moreover, the Isoflavones such as genistein and daidzein that are known for their estrogenic activity have shown a putative role in the prevention of breast cancer (Barnes 2003).

The subtropical fruit *C. betacea* belongs to the Solanaceae family (Lim 2013). Commonly known as tamarillo, tree tomato or ‘buah cinta’ among the locals. *C. betacea* is considered as an undervalue fruit in Malaysia, which is grown in Cameron Highland (Peninsular Malaysia) and Kundasang (Sabah). Several of underutilized fruits are reported to be rich in bioactive compounds which make them good potential sources for the production of neutraceuticals, flavors and pharmaceuticals (Ali Hassan and Abu Bakar 2013). However, these types of fruits are often underutilized due to their unidentified features, and economical prospective. Hence, these underutilized fruits provide unlimited opportunities for using as a potential phytomedicine as they are known to possess an array of chemical diversity, which needs to be investigated. There is a limited knowledge about the phytochemical profiles, antioxidant and anticancer potential of *C. betacea*. Hence the present study has been aimed to understand the antioxidant activities and antiproliferative effects of *C. betacea*. It has been reported that *C. betacea* is rich in β-carotene and ascorbic acid which makes them good natural sources of provitamin A and vitamin C. The fruits also having rich contents of anthocyanin and carotenoids which are responsible for their colour due to its natural pigments and both demonstrate the important biological, therapeutic, and preventative properties (de Rosso and Mercadante 2007).

Previous study reported that *C. betacea* displayed higher antioxidant properties than *Lycopersicon esculentum* (cherry tomato) and *Solanum lycopersicum* (tomato). The total phenolic content of C. betacea was 7.63 mg gallic acid equivalent/g (Noor Atiqah et al. 2014). Various research groups have screened a variety of fruit for their antioxidant activity but none appear to have included *C. betacea*. Thus, in the present study we aimed to determine the antiproliferative activity of *C. betacea* fruits against breast and liver cancer lines and also to study the polyphenol distribution.

## Methods

### Fruit sampling

The fruits of *C. betacea* were collected from Cameron Highland, Pahang, Malaysia, during June 2013. The herbarium specimens were identified and deposited in BORNEENSIS, University Malaysia Sabah, Malaysia. The fruits were cleaned and weighed. The small cut pieces were freeze-dried, and ground into fine powder using a grinder. The ground samples were sieved to get uniform size and then kept in an air-tight container and stored in a freezer (−20 °C) until further analysis.

### Sample preparation

Fine powders (2.5 g) were extracted with 50 ml 80% ethanol. The dry residue (50 mg) was dissolved in DMSO (1 ml) to obtain working solution of 1 mg/ml. These final extracts were filtered through a 0.45 µm nylon membrane syringe filter before use.

### MTT (3-(4,5-dimethylthiazol-2yl)-2,5-diphenyltetrazolium bromide) assay

The human tumour cell lines HepG2 (liver), MDA-MB-231(breast) and 3T3 (normal mouse fibroblast) cell lines were obtained from the American Type Culture Collection (ATCC, VA, USA). Cells were routinely grown with RPMI-1640 media supplemented with 10% fetal calf serum and 1% penicillin/streptomycin and incubated at 37 °C humidified atmosphere of 5% CO_2_ in T75 (75 cm^2^) flasks. The potential effects on cell viability were investigated using the MTT assay as an indicator of metabolically active cells. The cells were diluted in culture medium to a concentration of 1 × 10^5^ cells/ml and were pipetted into 96-well plates and incubated at 37 °C humidified, 5% CO_2_ incubator atmosphere to attach for 24 h. Cells were then exposed with *C. betacea* extracts (6.25, 12.5, 25, 50, 100, 200 µg/ml) through twofold serial dilutions (Kaewpiboon et al. 2014). Cells were also treated with doxorubicin (0.20, 0.39, 0.78, 1.56, 3.13, 6.25, 12.5, 25 µg/ml) that served as a positive control and culture medium (1% DMSO) was used as a negative control. After 72 h of extracts and drug exposure, the culture medium was removed and 10 μl of MTT reagent was added. Following incubation for 4 h, the MTT/medium was removed and DMSO (100 μl) was added to dissolve the formazan crystals. Absorbance of the colored solution was measured on an ELISA plate reader. Percentage of cytotoxicity was calculated as follows:

$$\% \;{\text{Cytotoxicity}} = \frac{{{\text{OD}}\;{\text{sample}}}}{{{\text{OD}}\;{\text{negative}}\;{\text{control}}}} \times 100\%$$

All experiments were performed in triplicate. The concentration of substance required for 50% growth inhibition (IC_50_) was estimated as that giving a 50% decrease in absorbance as compared to controls incubated simultaneously without substances.

### Determination of apoptosis by acridine orange/propidium iodide (AO/PI) staining

HepG2 and MDA-MB-231 cancer cell lines were seeded into 6 well plate at a density of 1 × 10^5^ cells/ml and incubated for 24 h at 37 °C in 5% CO_2_ incubator. After 24 h, cells were treated with *C. betacea* extract (IC_50_ values), doxorubicin (8 µg/ml) that served as positive control and culture medium (1% DMSO) that served as negative control for 24, 48 and 72 h. After exposure of grown cells in the extract, cells were washed with phosphate buffer saline (PBS) and harvested by centrifugation (2000 rpm for 5 min) and washed twice with PBS. The pellet (10 µl) were mixed with 5 µl (10 µg/ml) acridine orange and 5 µl (10 µg/ml) PI and observed under fluorescence microscope (Ekowati et al. 2010).

### DNA ladder assay

Fragmentation of chromatin DNA is one of the hallmarks of apoptosis. The quality of DNA was obtained with DNA laddering kit following manufacturer’s instructions Cayman Chemical (MI, USA). HepG2 and MDA-MB-231 (1 × 10^5^ cells/ml) cancer cells were seeded into 6-well plate and incubated for 24 h. After 24 h, HepG2 and MDA-MB-231 cells were treated with 30 and 80 µg/ml of *C. betacea* extracts, doxorubicin (8 µg/ml) and culture medium (1% DMSO) that served as negative control. After 72 h exposure of grown cells in the extract, cells were washed with phosphate buffer saline (PBS) and harvested by centrifugation at 2000 rpm for 5 min at room temperature (25 °C). The supernatant was discarded and pellet (1 × 10^5^ cells) in 200 µl of samples volume was mixed with 100 µl of Lysis Buffer and vortexed for 10 s. Then, the mixture was centrifuged at 8000 rpm for 1 min at room temperature. The supernatant was collected and mixed with 20 µl of 10% SDS for each sample and vortexed again before added 20 µl of Enzyme A. Samples were vortexed and incubated at 56 °C for 1 h. Then, 20 µl of Enzyme B was added to each sample and incubated at 37 °C for another 1 h. Precipitating reagent (130 µl) and ice-cold ethanol (950 µl) were then added to each tube. Samples were vortexed and centrifuged at 12,000*g* for 5 min at 4 °C. After centrifugation, supernatant was discarded and pellet was washed once with ice-cold 80% ethanol. Pellets were then dried on the bench for 5–10 min. TE buffer (100 µl) was added in each sample by vigorous vortexing. Samples (15 µl) were mixed with 3 µl of Gel-Loading Buffer (6×) and electrophoresed on a 1% agarose gel pre-stained with ethidium bromide at 75 V for 60 min. The fragmented inter-nucleosomal DNA was visualized using FluorChem 5500 Chemiluminescent (Alpha Innotech, CA, USA).

### Determination of polyphenol by HPLC

*HPLC apparatus*: The polyphenolic composition of the *C. betacea* fruits were determined by using HPLC–DAD (Agilent 1100, CA, USA). A reversed-phase C18 column (Nova-Pak, 150 mm × 4 mm, 5 µm) from Waters (MA, USA) was used throughout this study. All reagents were obtained from Merck (Darmstadt, Germany) HPLC grade. The prepared mobile phase was degassed using ultrasonic agitation and filtered under vacuum through a 0.45 µm nylon membrane filter before analysis. The rate flow was set at 1 ml/min. The operating temperature was maintained at room temperature (25 °C).

Analysis of phenolic acids and flavonoids: The polyphenolic composition of the *C. betacea* fruits was determined according to method as described by Uddin et al. (2014) with slight modification. Samples (1 g) were mixed with 4 ml of 80% ethanol for 24 h. The mixture was later centrifuged (5000 rpm for 15 min) at room temperature (25 °C). The supernatant was filtered through 0.45 µm nylon membrane filter prior use. The mobile phase was composed of (A) 0.5% acetic acid, and (B) methanol, and the gradient elution was performed as follows: 0 min, 100:0; 20 min, 10:90; and 30 min, 100:0 and filtered under vacuum through 0.45 µm membrane filter before use. The detection and quantification of gallic acid and caffeic acid was done at 254 nm, chlorogenic acid, epigallocatechin gallate, vanillic acid, p-coumaric acid and naringin was done at 280 nm, trans-ferullic acid and ferullic acid at 329 nm while quercetin and kaempferol were detected at 370 nm.

Standards: All standards were dissolved in 80% ethanol and prepared between the range of 20 and 100 µg/ml. All standards used were of the highest purity (HPLC grade) were obtained from Sigma (Darmstadt, St. Louis), were filtered through 0.45 µm nylon membrane syringe filter before injections.

### Statistical analysis

Data are presented as mean ± S.E.M and all experiments were carried out with three replicates. The data was statistically analysed using SPSS version 21.0. One-way analysis of variance (ANOVA) with Tukey’s test was used to test for differences between multiple groups. Level of significance was set at *p* ≤ 0.05. Independent-Samples *T* Test was used to test for significant differences in phenolic compounds of both extracts which was considered at the level of *p* ≤ 0.05.

## Results and discussions

### Cytotoxicity activity

**Fig. 1:**
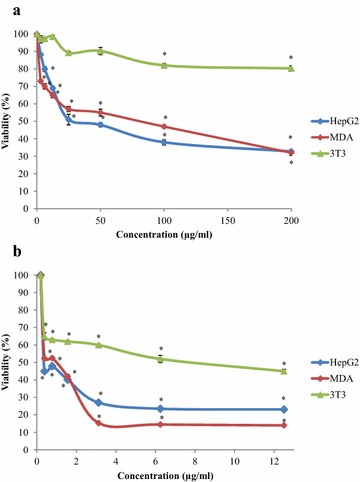
Cytotoxic effects of *C. betacea* (**a**) and doxorubicin (**b**) on HepG2 and MDA- and 3T3 cell lines viability, assessed by MTT assay. Values are presented as means (n = 3) ± SD, and *p < 0.05 compared to negative control

The cytotoxic behaviour of *C. betacea* fruits were tested in two different human cancer cell lines: liver (HepG2) and mammary gland (MDA-MB-231) adenocarcinoma. The experiments were also carried out in normal mouse fibroblast (3T3) to investigate its toxicity towards normal cells. The endpoint criteria for antiproliferative activity was selected according to the cytotoxic activity of IC_50_ values that should be below 100 µg/ml for crude extracts and less than 25 µM for pure compounds (Cos et al. 2006). Figure 1a shows that *C. betacea* extract strongly inhibited the proliferation of HepG2 and MDA-MB-231 cell lines in a dose-dependent manner with IC_50_ values of 30 and 80 µg/ml, respectively. It was verified that the increased in concentration of *C. betacea* fruits leads to a higher cytotoxic activities. However all the sample extracts did not exert any significant cytotoxic effect against 3T3 cell (IC_50_ > 200.00 µg/ml). Doxorubicin (chemotherapy drug) was capable to induce cytotoxicity in HepG2 and MDA-MB-231 with IC_50_ value of 0.35 and 0.78 µg/ml respectively, as shown in Fig. 1b. Nevertheless, results also proved that doxorubicin showed superior cytotoxic activity against 3T3 normal cell with IC_50_ value of 8 µg/ml (Fig. 1b).

Previous study reported that the cancer preventing action of fruits and vegetables is most probably due to the several bioactive compounds that act simultaneously to prevent cancer rather than being one or two potent anticarcinogens (Tomás-Barberán and Gil 2008). As chemopreventive agents, phytochemicals can interfere in different step of carcinogenesis (promotion, initiation and progression) by two major actions; cancer blocking agents and cancer suppressing agent (Surh 2003). Some chemopreventive phytochemicals inhibit the metabolic action of the pre-carcinogens, consequently block the tumor initiation.

### AO/PI double staining and fluorescent microscopy

**Fig. 2:**
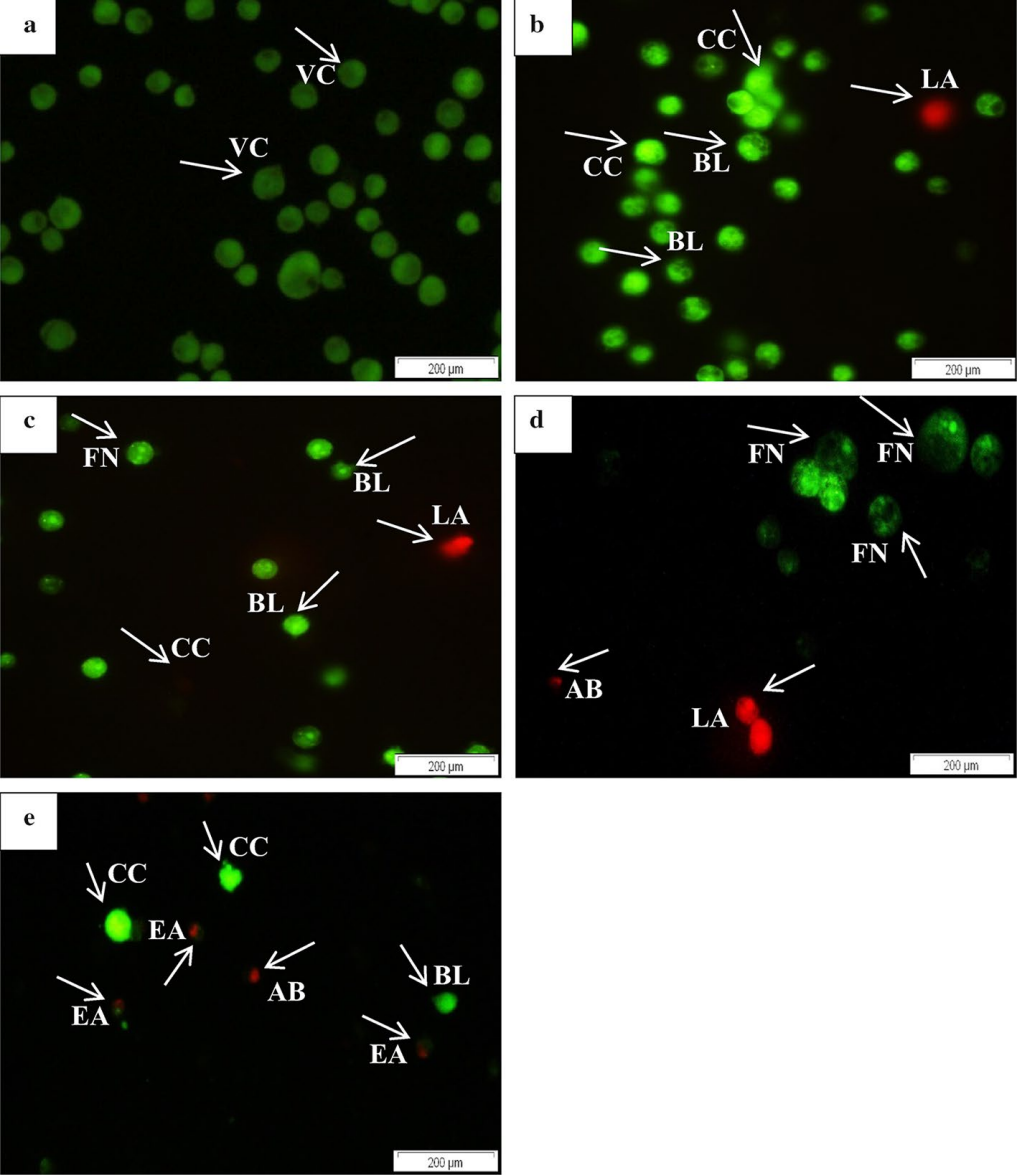
Fluorescent micrographs of AO/PI double stained of HepG2 cells treated with *C. betacea* at 30 µg/ml for 24, 48 and 72 h. **a** Untreated HepG2 cells showing normal cell structure and viable cells fluoresce green with round intact nuclei. **b** After 24 h treated group showing chromatin condensation, membrane blebbing and late apoptosis. **c** Chromatin condensation, membrane blebbing, DNA fragmentation and late apoptosis events were observed after 48 h of treatment. **d** Apoptotic body formation after 72 h of treatment. **e** HepG2 cell treated with 8 µg/ml of doxorubicin showed early apoptosis event and formation of apoptotic body. *Scale bars* (A–E): 200 μm (magnification: × 200). *VC* Viable cells, *BL* Blebbing of cell membrane, *CC* Chromatin condensation, *FN* Fragmented nuclei, *EA* Early apoptosis, *LA* Late apoptosis, *AB* Apoptotic bodies

**Fig. 3:**
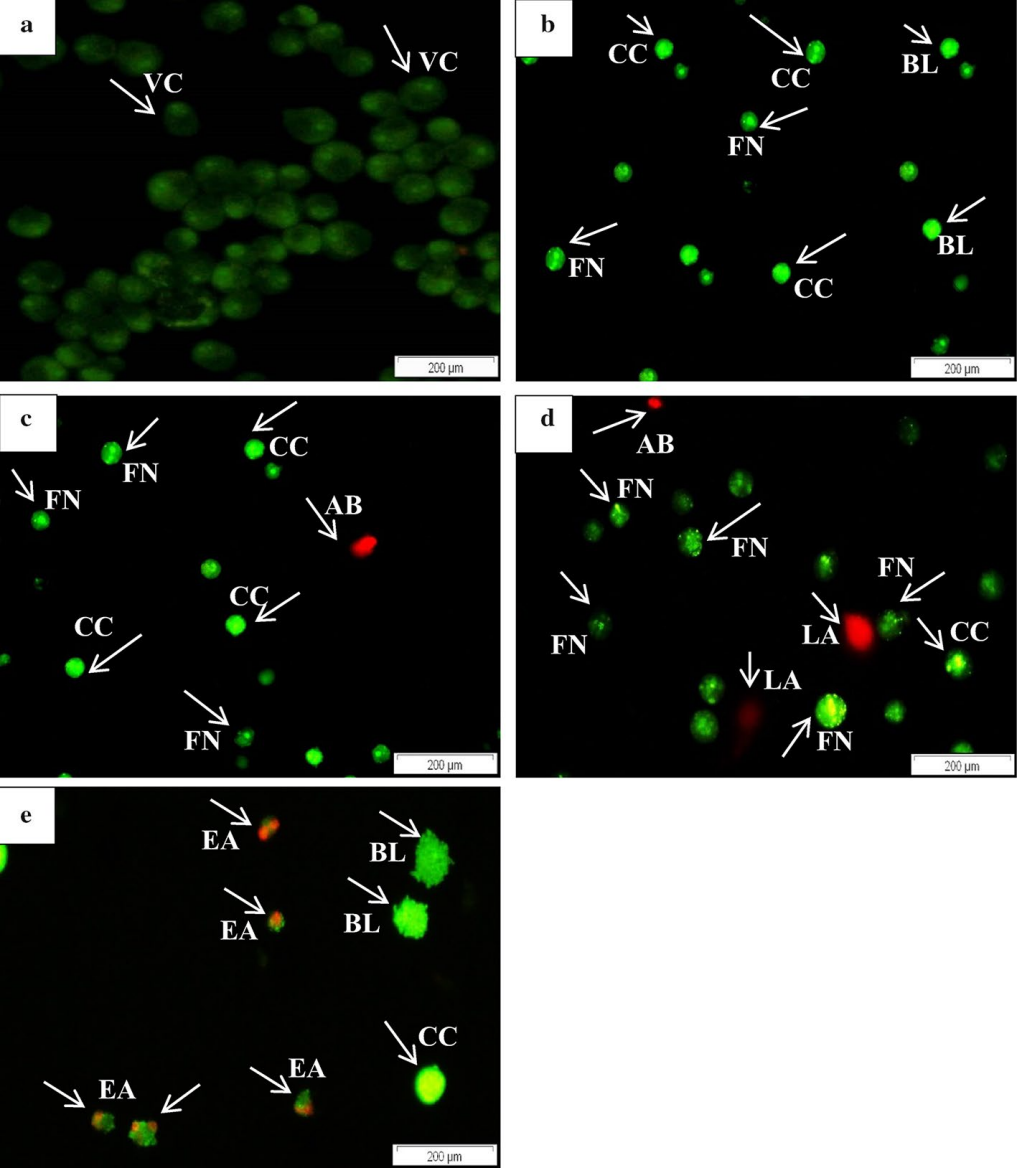
Fluorescent micrographs of AO/PI double stained of MDA-MB-231 cells treated with *C. betacea* at 80 µg/ml for 24, 48 and 72 h. **a** Untreated MDA-MB-231 cells showing the normal viable cells with circular nucleus uniformly distributed in the centre of the cell. **b** Blebbing or signs of membrane protrusions and fragmented of nuclei were evident after 24 h of treatment. Nucleus showed green fluorescence and concentrated into a granular that indicates chromatin condensation. **c** 48 h treated cells detected as rounding off, reduction in cell size, damaged membrane and appearance of apoptotic body. **d** Necrotic cells: 72 h treated cells showed late apoptosis events with formation of apoptotic body, unsymmetrical shape and size, uneven and disintegrated membrane surface. **e** MDA-MB-231 cell treated with 8 µg/ml of doxorubicin showed early apoptosis events with appearance of membrane blebbing and chromatin condensation. *Scale bars* (**a**–**e**): 200 μm (magnification: × 200).*VC* Viable cells, *BL* Blebbing of cell membrane, *CC* Chromatin condensation, *FN* Fragmented nuclei, *EA* Early apoptosis, *LA* Late apoptosis, *AB* Apoptotic bodies

Staining cells with fluorescent dyes is useful in evaluating the nuclei morphology of apoptotic cells and mode of cell death. Apoptotic and viable cells of HepG2 and MDA-MB-231 were evaluated using fluorescent microscope. Healthy viable cells of HepG2 and MDA-MB-231 were visualized well rounded, large and intact green nuclear structure (Figs. 2a, 3a) while observations of cells treated with *C. betacea* extract based on their IC_50_ values were identified shrinking with condensed fragmented nuclei that confirmed apoptosis in a time dependent manner (Figs. 2b–d, 3b–d). Early apoptosis features (Figs. 2e, 3e) were seen for HepG2 and MDA-MB-231 treated with 0.35 and 0.78 µg/ml of Doxorubicin, respectively. AO penetrated within the fragmented DNA (FN) and displayed nuclear condensation that indicates moderate apoptosis. Late stage of apoptosis (LA) showed the presence of apoptotic bodies (AB) observed as orange-red fluorescence represents the hallmark of late apoptosis (Figs. 2b–d, 3c, d). In addition, cells were noticeable with asymmetrically localized, membrane blebbing (BL) and nuclear margination (CC) as can be seen in Figs. 2b–e and 3b–e for HepG2 and MDA-MB-231, respectively.

### DNA laddering

**Fig. 4:**
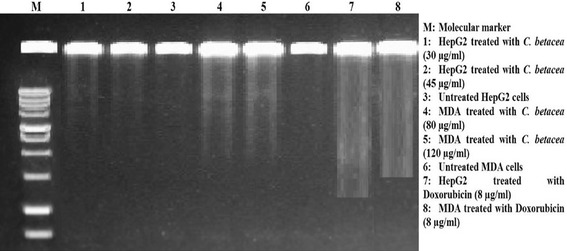
DNA ladder assay. *Lane M* Molecular marker 100 bp; *Lanes 1* and *2*: HepG2 cell line treated with *C. betacea* extract at 30 and 45 µg/ml, respectively; *Lanes 4* and *5* MDA-MB-231 cell line treated with *C. betacea* extract at 80 and 120 µg/ml, respectively; *Lanes 7* and *8* HepG2 and MDA-MB-231 cell lines treated with doxorubicin (8 μg/ml), respectively; *Lanes 3* and *6* untreated HepG2 and MDA-MB-231 cell lines, respectively

Detection of DNA fragmentation become a widely accepted biochemical hallmark of apoptotic cytotoxicity and drug induced apoptosis in cancer cells (Kroemer et al. 1995). Figure 4 shows the DNA fragmentation which was detected in HepG2 (lane b and c) and MDA-MB-231 (Lane D and E) cancer cell lines treated with *C. betacea* extract based on their IC_50_ and IC_75_ values after 72 h incubation. However, there is no DNA fragmentation was observed in control (untreated) cells as shown in Lane D and Lane G for HepG2 and MDA-MB-231 cells, respectively. Conversely, the standard doxorubicin induced the fragmentation of the DNA at very low concentration (8 µg/ml) as shown in Lane H and Lane I for HepG2 and MDA-MB-231 cells, respectively. These results indicate that cytotoxic activity is present in *C. betacea* and exerted substantial DNA damage leading to apoptotic cell death in HepG2 and MDA-MB-231 cancer cell lines.

### Phytochemical compositions

#### Phenolic acids composition

The major secondary metabolites in plants include terpenoids, alkaloids, sulphur-containing compounds and phenolic compounds (Dillard and Bruce 2000). The main phenolic subclass in fruits are phenolic acids (hydroxybenzoic and hydroxycinnamic acid), flavonoids, and coumarin (Hounsome et al. 2008). Phenolic acids in general are phenols with one carboxylic acid group. Hydroxybenzoic acids (vanillic and gallic acid) have the carboxylic acid group directly attached to the ring, while hydroxycinnamic acids (*p*-coumaric acid, caffeic acid, ferulic acid, *trans*-ferulic acid and chlorogenic acid) have a three-carbon side chain. Phenolic compounds have been reported to possess antioxidants, antibacterial, anticancer and anti-inflammatory properties (Ghasemzadeh et al. 2010).

**Fig. 5:**
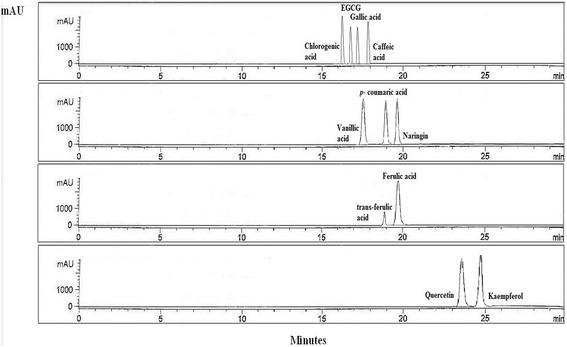
HPLC chromatogram of the *C. betacea* extract detected at 254, 280, 329 and 370 nm. Peaks: 1 = gallic acid; 2 = caffeic acid; 3 = vanillic acid; 4 = *p*-coumaric acid; 5 = naringin; 6 = trans-ferulic acid; 7 = ferulic acid; 8 = kaempferol

**Fig. 6:**
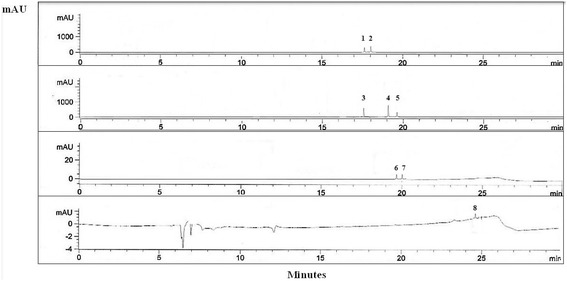
HPLC chromatogram of the flavonoid and phenolic acid standards at 254, 280, 329 and 370 nm

Figure 5 illustrates the separation of flavonoids and phenolic acids in *C. betacea* extract, while Fig. 6 shows the mixture of 11 external standards for flavonoids and phenolic acids. A good separation was achieved in a short separation time of 26 min. Phenolic acid contents achieved from *C. betacea* extract were presented in Table 1. Out of seven phenolic acids tested, gallic, caffeic and vanillic acids were found to be most abundant in the samples tested. While ferulic, p-coumaric and trans-ferulic acids were found in minor quantity in the samples tested ranged from 0.05 to 0.49 µg/g DW. Caffeic and gallic acids display a wide variety of biological functions including their primary antioxidant activity, which are mainly related to the modulation of carcinogenesis. In addition, gallic acid has been proven to protect against oxidative damage induced by reactive oxygen species (ROS) and act as chemopreventive agents that cause apoptosis in the proliferation of several tumour cell lines. For an instance, Lim (2013) has reported that *C. betacea* prevented oxidative stress-induced cell death in HepG2 cells in a dose-dependent manner.

**Table 1:** Phenolic acid compositions (µg/g DW^a^) of *C. betacea*

Samples	µg/g DW^a^	RT (min)	Area (mAU)
Ferulic	0.05	19.630	31.44
*p*-coumaric	0.41 ± 0.47	19.578	38.86
Caffeic	1.65 ± 0.72	17.677	28.25
Vanillic	1.11 ± 1.82	17.682	15.99
Trans-ferulic acid	0.49 ± 0.51	19.622	32.35
Gallic	3.02 ± 1.36	17.676	26.99

^a^Dry weight; data are presented in mean ± S.E.M (*n* = *3*); N.D. - not detected

The genus of *Solanum* (Solanaceae) can be an important source of natural antioxidants, particularly the phenolic acid compositions. Some of these phytochemicals include 2-methyl[1,3,4]oxadiazole, 2,3-dihydro-3,5-dihydroxy-6-methyl-4*H*-pyran-4-one and thiazole that may have the role in anti-inflammatory effects while n-hexadecanoic acid may be responsible in the antioxidant activities. The other compound identified was 1,3,4-Oxadiazole (OXD) products that are well-known for their anti-inflammatory, (Dr. Duke’s phytochemical and ethnobotanical databases), antibacterial, antifungal activity (El-Emam et al. 2004) and HIV replication inhibition (Sahin et al. 2002). Furthermore, thiazole was also reported previously to have anticancer properties which suggest the potential of the medicinal activities of tamarillo fruits. It was reported that the hydroxycinnamic acid derivatives is dominant in the peel and pulp of *C. betacea* (Vasco et al. 2009). Noor Atiqah et al. (2014) found that *C. betacea* contain considerable high mean of total phenolic content (7.63 ± 0.37 mg GAE/g). The same study also reported there was also a positive correlation existed between antioxidant activity and total phenolic content of *C. betacea* which suggest that the predominant source of antioxidant potential of *C. betacea* derives from the polyphenolic compounds present in the fruits. Overall, the data suggested that *C. betacea* may be a good source of antioxidant compounds in nutraceutical or functional food products.

### Flavonoids composition

Flavonoids are the most diverse group of secondary metabolite that involve in plant growth, reproduction, seed germination and protection against pathogen (Agati et al. 2012). The structural subclass of flavonoids depend on the modifications of the C-ring and they can be divided into; flavonol (quercetin, rutin and kaempferol), flavone (apigenin, luteolin), flavan-3-ol (epicatechin, catechin), anthocyanin (delphinidin and cyaniding), isoflavone (genistein and diazein) and flavanone (hesperetin and naringenin). Flavonoids are the most studied phenolic compounds, due to their biological effects including antioxidant, antimutagenic, anticarcinogenic, and antibacterial properties (Kumar and Pandey 2013). The amount of selected polyphenols (flavonols and flavanones) in *C. betacea* were reported in Table 2.

**Table 2:** Flavanoids composition (µg/g DW^a^) of the fruits

Samples	µg/g DW^a^	RT (min)	Area (mAU)
Flavonols			
Quercetin	N.D.	N.D.	N.D.
Kaempferol	0.5 ± 0.80	24.693	13.17
Flavanones			
Naringin	3.32 ± 1.50	19.582	37.95

^a^Dry weight; data are presented in mean ± S.E.M (*n* = *3*); N.D. - not detected

#### Flavonols

Flavonols compound such as quercetin and kaempferol in *C. betacea* were examined and shown in Table 2. We detected kaempferol at lower levels with the values of 0.5 ± 0.80 µg/g DW in *C. betacea* however, quercetin was not detectable. Tokuşoğlu et al. (2003) reported that kaempferol levels in *S. esculentum* (tomato) which share the same genus with *C. betacea* were identified in the range between 0.2–0.6 µg/g FW, which were in accordance to our findings. Food derived flavonoids particularly flavonols (kaempferol, quercetin, and myricetin) are reported to exhibit multiple biological functions such as antioxidant, anti-allergenic, anti-inflammatory, cardio-protective and vasodilatory effects. In addition to all these activities, flavonols also has strong anticancer activity on several cancer cells such as leukaemia, breast, ovarian, gastric and liver (Joshi et al. 2011).

#### Flavanones

Table 2 shows the amount of flavanones (naringin) present in *C. betacea* fruits with the values of 3.32 ± 1.50 µg/g DW. Flavanones accounted for 90-98% of flavonoids in citrus fruits such as sweet oranges, lemons, limes and grapefruit (Fowler and Koffas 2009). Flavanones have been demonstrated to induce cytotoxic activity toward various human cancer cell lines with little effect on normal cells. Patil et al. (2009) found that lime juice rich in hesperidin inhibits human pancreatic cancer cell, while Harmon and Patel (2004) reported that the regular intake of flavanones inhibits proliferation of breast cancer cell. This suggests that *C. betacea* fruit with moderate amount of flavanones content making them ideal candidates in developing the potential flavonoid-based chemotherapeutics for anticancer treatment.

## Conclusions

In conclusion, the present study demonstrated that the crude extract of C. betacea possessed antiproliferative activity against HepG2 and MDA-MB-231 cancer cell lines, and more importantly did not exerted an inhibitory effect on the proliferation of the normal cells (3T3). The profiling of phenolic acids and flavonoids by HPLC-DAD-MS has identified some of the major polyphenols that are likely to contribute to the chemopreventive activity. Previous study has found that *C. betacea* exhibited high antioxidant activity largely derived from polyphenolic, flavonols and anthocyanins compounds. It can be concluded from this study that *C. betacea* has a surprisingly wide range of beneficial properties including antioxidant and anticancer properties and has good potential to be further developed in nutraceutical or functional-food products.

## Abbreviations

MTT: 3-(4,5-dimethylthiazol-2yl)-2,5-diphenyltetrazolium bromide)
HPLC: high performance liquid chromatography
IC_50_: concentration to inhibit cellular proliferation by 50%
DMSO: dimethyl sulfoxide
HepG2: liver hepatocellular carcinoma
MDA: homo sapiens mammary gland
3T3: normal mouse fibroblast
CO_2_: carbon dioxide
ELISA: enzyme-linked immunosorbent assay
OD: optical density
PBS: phosphate buffered saline
PI: propidium iodide
HPLC–DAD-MS: high-performance liquid chromatography-diode array detection-mass spectrometry
SPSS: statistical package for the social sciences
ANOVA: analysis of variance
AO/PI: acridine orange/propidium iodide
AO: acridine orange
DNA: deoxyribo nucleic acid
GAE: gallic acid equivalent
ROS: reactive oxygen species
FW: fresh weight
DW: dry weight
EGCG: epigallocatechin 3-gallate
SEM: standard error of the mean
